# The Communicability of Phthisis

**Published:** 1869-03

**Authors:** 


THE COMMUNICABILITY OF PHTHISIS.—In a letter to the editor of the *Medical Times and Gazette*, R. P. Cotton, M. D., Senior Physician to the Hospital for Consumption, Brompton, says: "My attention has just now been directed to an abstract of a paper by Dr. Elliott upon the communicability of phthisis, in the last number of last year's *Medical Times and Gazette.* Dr. Elliott appears to be a firm believer in Dr. Budd's theory of the zymotic character of consumption, and supports his views by asserting 'the frequency of phthisis amongst the nurses of the Brompton Hospital.' As such a statement (given, I would observe, without any authority,) is opposed to fact, and the question is of grave importance, I hope you will allow me, although somewhat late in doing so, to refer Dr. Elliott and those of your readers interested in the subject to a detailed report of mine on this very matter published in the *Lancet* of November 2d, 1867, as a reply to Dr. Budd's views. It will there be seen that the nurses and other resident and non-resident officials connected with the Hospital for Consumption at Brompton have been always remarkably free from phthisis, affording in this respect very evident and strong testimony in favor of the non-communicability of the disease."—*Boston Medical Journal.*

